# Time to development of adverse drug reactions and associated factors among adult HIV positive patients on antiretroviral treatment in Bahir Dar City, Northwest Ethiopia

**DOI:** 10.1371/journal.pone.0189322

**Published:** 2017-12-21

**Authors:** Etsegenet Kindie, Zelalem Alamrew Anteneh, Endalkachew Worku

**Affiliations:** 1 Yinesa Health Center, Bahir Dar District, Bahir Dar, Ethiopia; 2 School of Public Health, College of Medicine and Health Sciences, Bahir Dar University, Bahir Dar, Ethiopia; Istituto di Genetica Molecolare, ITALY

## Abstract

**Background:**

Adverse drug reactions (ADRs) are harmful and unintended reactions to medicines given at standard doses. Although the antiretroviral treatment (ART) changed the global HIV epidemic significantly, it’s associated adverse events is huge. Therefore, investigating the rate and development of ADRs of ART provides vital information for monitoring risks.

**Methods:**

Retrospective study was conducted among patients on ART from July1/2011—June 30/2016 at Felege Hiwot referral hospital. Data were collected using checklist and document review. The p-value and hazard ratio with its confidence interval was used to show presence and strength of association.

**Results:**

A total of 602 subjects were studied. The rate of occurrence of major ADRs was 4.3/100PY. Patients with no formal and completed primary education were at higher risk of developing ADRs compared to those with higher level education [AHR = 8, 95% CI: 2.53–25.20, AHR = 4.9, 95% CI: 1.65–14.44]. The risks of ADRs among patients working in NGOs were more than four times compared to those in governmental organizations [AHR = 4.3, 95% CI: 1.42–13.31]. The risks of ADRs in WHO clinical stage II, III and IV were much higher than in stage I [AHR = 4, 95% CI: 1.33–11.93, AHR = 5.3, 95% CI: 2.02–13.79 and AHR = 7, 95% CI: 2.51–20.10] respectively. Moreover, patients didn’t receive OI prophylaxis were more three times at risk of ADRs compared to those received [AHR = 3.2, 95% CI: 1.47–7.08].

**Conclusions:**

Most of the ADRs cases were occurred within a year after initiation of ART. Educational status, occupation, advanced clinical stage and OI prophylaxis therapy were predictors ADRs. Continuous counseling for non-educated patients and clients in clinical stage II and above, and patients didn’t take OI prophylaxis need to get close follow up to prevent the associated ADRs by the concerned parties.

## Introduction

Adverse drug reactions (ADRs) are harmful and unintended reactions to medicines given at standard doses through a proper route of administration for the purpose of prophylaxis, diagnosis, or treatment. All drugs carry the potential to produce both desirable and undesirable effects [1]. Continuous surveillance of safety and efficacy of the pharmaceutical products is an essential part of the patient care system in clinical practice. Continuous evaluation of the benefits and harms of drugs will help to achieve the ultimate goal to make safer and more effective treatment available to patients [2].

This days, antiretroviral treatment (ART) is changing the global HIV epidemic in significant ways, and the scaling up of ART averted about 4.2 million deaths in low- and middle-income countries in 2002–2012 [3].However, ART-associated adverse events can range from acute and potentially life threatening to chronic and insidious disorders; these conditions often require an immediate discontinuation of all antiretroviral (ARV) drugs and re-initiation of an alternative regimen without overlapping toxicity [4].

Adherence to ART depends on a number of factors but the most important factor was the type and severity of adverse drug reactions experienced by the patients. Adverse drug reactions are the single most common reason for poor adherence to treatment [4, 5] Evidences showed that up to 25% of patients discontinue their initial highly active antiretroviral therapy (HAART) regimen because of toxic effects [6].High frequency of regimen discontinuation and switch are observed in patients who experienced anemia, rash, hepatotoxicity, and peripheral neuropathy[7]. The incidence rate of treatment modification was 18.64 per 100 person years over 946 person years of follow-up. The rate of modification was higher in the first year of ART compared to second and the third year[8]. The overall ADR incidence rate was 9.5% in Malawi[9].

The prevalence of ADRs is variably reported from different studies which are 65.5% in Ethiopia, 83% in Zimbabwe and 75.4% in central India [10, 11]. In India, almost 37.70% of PLWHAV develop ADRs within 0–6 month’s duration of treatment followed by 30% PLWHAV develop within the duration of 6–12 months [5].As ADRs have the potential to cause significant harm in patients, there is a need to increase awareness on the impacts of ADRs to patient care and public health. Local factors including the high prevalence of HIV/AIDS, tuberculosis, health systems failures and illiteracy among patients contribute to the burden of drug related morbidity and mortality in Ethiopia [12]

“Serious” reactions include those that result in death, are life-threatening, result in hospitalization or prolongation of hospitalization or result in permanent harm or disability. Adverse reactions that result in treatment discontinuation and a change in ART regimen are also monitored as serious [13]. In resource limited setting including Ethiopia, where treatment options are limited; information about the development of adverse reaction of ART is vital for monitoring the risks. However, there was limited information on the subject in Ethiopia. Therefore, the aim of this study was to determine the incidence rate and time to the development of adverse reaction of first line HAART regimen. The study provides pertinent data for treatment guidelines review, regulatory authority for control, pharmaceutical planning &decision makings

## Materials and methods

The study was conducted at Felege Hiwot referral hospital in Bahir Dar City, North West Ethiopia. The study used data collected from July 1/2011 to June 30/2016 retrospectively. All adult HIV positive patients on ART who started first line ART between July 1/2011 to June 30/2016were our study population. The record lists of all HIV positive adult patients who commenced first line HAART between these periods were used as a sampling frame. Out of the total 2374 adult HIV patients on initial regimen, 602 patients were selected by systematic random sampling method. Every 4^th^ in the list were included the study.

### Measurement of the outcome variable

According to this study an adverse drug reaction (ADR) had occurred if a patient reported at least one of the following events: hospitalization, or switch/discontinued drug because of adverse drug reaction. The available information on the card was observed and data were collected using data extraction checklist. The check list was prepared based on the variables available on the card and reviewed literature sources [6–8, 10].

Five data collectors and one supervisor who had ART training and similar data collection experiences were recruited, and one day training was given for them on the objectives of the study, sampling methods and research ethics. The checklist was pretested on 30 HIV positive patient cards in Bahir Dar health center to check for any amendment.

The collected data were checked for its completeness, coded and entered in to Epi Info version 7 and analyzed using SPSS software package version 21. Descriptive statistics including frequencies and percentages were carried. The incidence density of the occurrence of ADRs was calculated.

Kaplan-Meier survival curves were used to show the association between covariates and the timing of ADRs, and to describe the differences in the survival rates. Both bivariate and multivariable Cox proportional hazard models were used to identify explanatory variables. Variables with p- value 0.2 and less in the bivariate analysis were entered into the multivariable proportional hazard model.

P-values less than 0.05 and the corresponding hazard ratio with its 95% confidence intervals (CI) in the multivariable Cox proportional hazards model were considered as significantly associated with the time of ADRs.

Ethical clearance was obtained from Bahir Dar University Ethical review committee and permission letters were obtained from Amhara Regional Health Bureau and Felege Hiwot Referral Hospital administration offices, and all these parties approved the study. The patients′ clinical records were reviewed anonymously and confidentiality of the data was kept at each step of data collection and processing.

## Results

### Socio demographic characteristics

A total of patient 602 records were analyzed. The mean and standard deviation of the age of the clients at the initiation of ART was 35.05 ± 9.6 years. Two hundred fifty four (42.2%) of the participants were in the age group between 25 and 34 years. Three fifth of the participants (59.1%) were females. Regarding the level of education, 175 (29.1%) had no formal education and 152(25.2%) were in primary school education (See Table 1).

**Table 1:** Socio demographic characteristics of HIV positive adults at initiation of HAART at Felege Hiwot Referral Hospital, July 2011 to June 2016 (n = 602).

Variables	Category	Frequency	Percentages
Sex	Male	246	40.9
	Female	356	59.1
Age (years)	15–24	52	8.6
	25–34	254	42.2
	35–44	193	32.1
	≥45	103	17.1
Religion	Orthodox	544	90.4
	Muslim	46	7.6
	Protestant	10	1.7
	Others	2	0.3
Ethnic group	Amhara	597	99.2
	Tigray	5	0.8
Educational status	No formal education	175	29.1
	Primary school education	152	25.2
	Secondary school education	119	19.8
	Higher institute education	156	25.9
Marital status	Never married	99	16.4
	Currently married	320	53.2
	Divorced	148	24.6
	Widowed	35	5.8
Occupational status	Governmental employee	145	24.1
	Non- governmental employee	53	8.8
	Self employed	169	28.1
	Daily labourer	74	12.3
	House wife	130	21.6
	Others	31	5.1

### Clinical and immunological characteristics of the study subjects

Nearly two fifth (41.9%) of patients were on WHO stage III at the time of HAART initiation. The inter quartile ranges of CD4 count for the study participants was 188cells/μl, 55.1% of the study subjects had CD4 count less than 200 cells/μl.

The mean and standard deviation of the weight of the patients was 53.6 ±9.96; three fourth (75.9%) of the patients had a body weight of less than 60 kg. Majority of (85.5%) patients had received Cotrimoxazole prophylaxis and (7.5%) of the patients had TB co-infection.

The predominant HAART regimen initially prescribed for the patients were a combination of Tenofovir, Lamivudine and Efavirenz (TDF-3TC-EFV) 353(58.6%) followed by zidovudine, Lamivudine and Nevirapine (AZT-3TC-NVP) 137 (22.8%) (See Table 2).

**Table 2:** Clinical and immunological characteristics of HIV positive adults at initiation of HAART at Felege Hiwot Referral Hospital, July 2011 to June 2016 (n = 602).

variables	Category	Frequency	Percentages
WHO clinical stage	WHO stage I	162	26.9
	WHO stage II	138	22.9
	WHO stage III	252	41.9
	WHO stage IV	52	8.3
Weight (kg)	<60	457	75.9
	≥60	145	24.1
OI prophylaxis	Yes	517	85.8
	No	85	14.2
CD4count(**cells/μL**)	<200	332	55.1
	≥200	270	44.9
Functional status	Working	518	86
	Ambulatory	69	11.5
	Bedridden	15	2.5
TB co-infection	No	557	92.5
	Yes	45	7.5
Initial ART regimen	AZT + 3TC + NVP (1c)*	137	22.8
	AZT + 3TC + EFV (1d)**	66	11
	TDF + 3TC + NVP (1f)ǀ	42	7
	TDF + 3TC + EFV (1e)ǁ	353	58.6
	ABC+ 3TC + EFV (g)ǂ	4	0.7

*- zidovudine, lamivudine, nevirapine.

^ǀ^ - Tenofovir, lamivudine, nevirapine.

**- zidovudine, lamivudine, Efavirenz.

^ǁ^ - Tenofovir, lamivudine, Efavirenz.

^ǂ^ - abacavier, lamivudine, Efavirenz.

### Occurrence of adverse reactions

Out of 602 study participants followed retrospectively for the last five years; a total of 61 (10%) of the study subjects had experienced adverse drug reactions in 1435 person years (PY) of observations.

Of the 61 events of ADRs, 38 (62%) were drug changes/switches/, and the remaining 23 (37.8%) were hospital admissions. The overall occurrence rates of adverse drug reaction was 4.3/100 PY.

About 37(60.6%), 12(19.7%) and 9(14.75%) adverse drug reactions were occurred within the first half, one and two years of the initiation of ART respectively. The cumulative probability of survival without developing ADRs at the end of half, one and two years was 0.94, 0.91, and 0.89 years respectively. And the cumulative probability of survival by the end of the follow up period was found to be 0.88 (see Table 3).

**Table 3:** Life table for adverse drug reaction survival among adult HIV positive patients in Felege Hiwot Referral Hospital in 2017.

Interval Start Time	Number Entering Interval	Number Withdrawing during Interval	Number Exposed to Risk	Number of Terminal Events	Proportion Terminating	Proportion Surviving	Cumulative Proportion Surviving at End of Interval	Std. Error of Cumulative Proportion Surviving at End of Interval	Probability Density	Hazard Rate	Std. Error of Hazard Rate
0	602	0	602	0	0	1	1	0	0	0	0
1	602	102	551	49	0.09	0.91	0.91	0.01	0.089	0.09	0.01
2	451	100	401	9	0.02	0.98	0.89	0.01	0.02	0.02	0.01
3	342	101	291.5	1	0	1	0.89	0.01	0.003	0	0
4	240	137	171.5	2	0.01	0.99	0.88	0.02	0.01	0.01	0.01
5	101	101	50.5	0	0	1	0.88	0.02	0	0	0

### Association between explanatory variables and adverse drug reaction among HIV patients on ART

**Fig 1:**
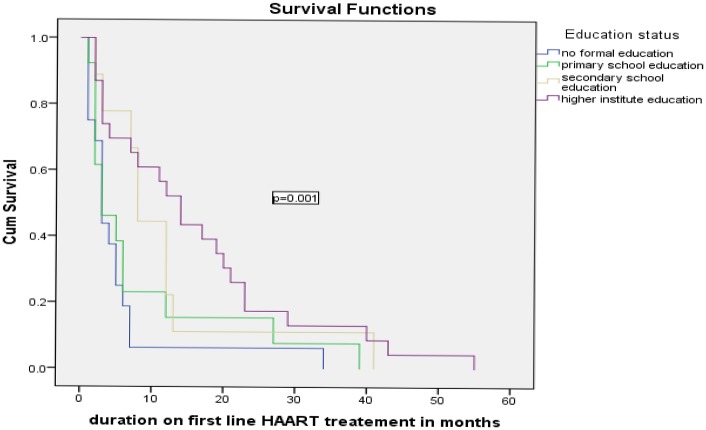
Kaplan Meier curves for time to the development of ADRs among HIV patients on ART, felge hiwot Referral hospital, 2011–2016 classified by educational status.

**Fig 2:**
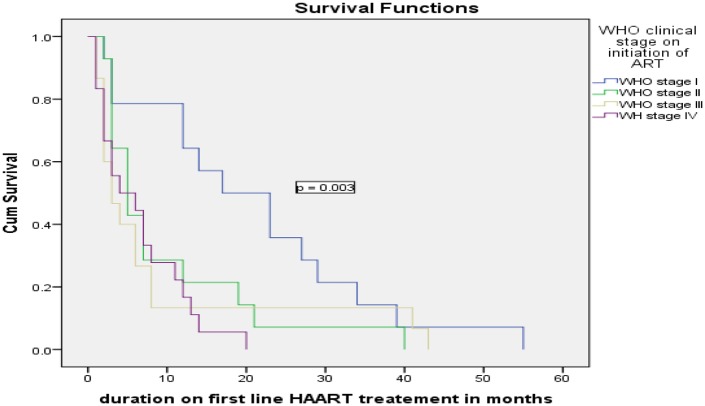
Kaplan Meier curves for time to the development of ADRs among HIV patients on ART, Felege Hiwot Referral hospital, 2011–2016 classified by WHO clinical stage.

**Fig 3:**
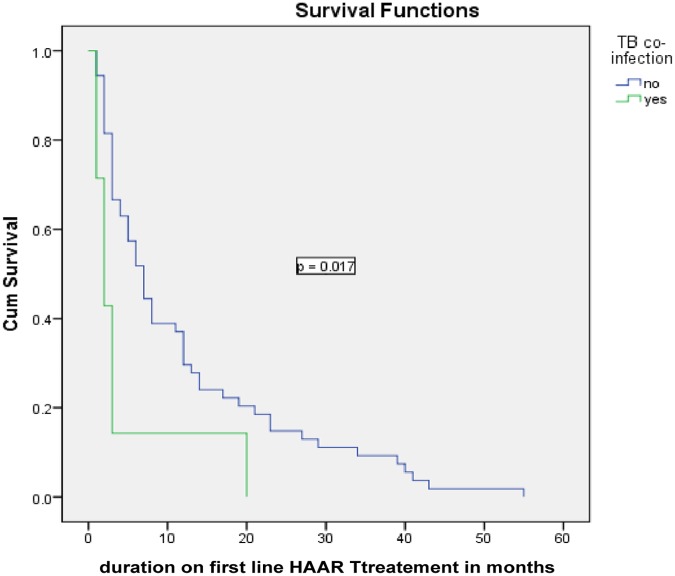
Kaplan Meier curves for time to the development of ADRs among HIV patients on ART, Felege Hiwot Referral hospital, 2011–2016 based on TB co-infection.

Kaplan-Meier survival curves were used to describe the association between predictor variables and timing of the ADRs in the survival rates. Educational status of the patients had a significant effect on timing of experiencing ADRs (log rank test = 0.001), non-educated patients had short survival time compared to those with higher education. Opportunistic infection (OI) prophylaxis drug intake had a significant effect on timing of experiencing adverse drug reactions (p = 0.017) (see Figs 1–3).

### Factors associated with time to the development of ADRs among HIV positive patients on ART

In the bivariate (crude) Cox-regression analysis, educational status, occupation, baseline WHO clinical stage, TB co-infection, OI prophylaxis intake and CD4 count were showed association at P-value of less or equal to 0.2. The initial ART regimen was not showed association with the adverse drug reaction. The variables with p-value 0.2 or less in the bivariate result were entered in to multivariable Co-regression analysis.

Accordingly, educational level of the clients showed a significant association Patients with no formal education and completed primary education at initiation of HAART had higher risk of developing adverse drug reaction compared to those with higher educational level [p = 0.001, AHR = 8, 95% CI: 2.53–25.20, AHR = 4.9, 95% CI: 1.65–14.44] respectively.

Patients who were working in non-governmental organization were at higher risk of developing adverse drug reactions as compared to those working in governmental organizations [p = 0.01, AHR = 4.3, 95% CI: 1.42–13.31].

Patients with WHO clinical stage II, III and IV were higher risk developing ADRs than patients whose clinical stage was I respectively [p = 0.001, AHR = 4, 95% CI: 1.33–11.93, AHR = 5.3, 95% CI: 2.02–13.79 and AHR = 7, 95% CI: 2.51–20.10].

Moreover, patients didn’t receive OI prophylaxis were more likely to develop ADRs compared to their counter parts [p = 0.004, AHR = 3.2, 95% CI: 1.47–7.08] (See Table 4).

**Table 4:** Cox regression analysis between different predictor variables and time to the development of ADRs among adult HIV positive patients on HAART regimen at Felege Hiwot Referral Hospital, July 2011 to June 2016 (n = 602).

Variables	Survival status		CHR (95%CI)	AHR (95% CI)	P-value
	Event	censored			
**Educational status**					
No formal education	16	159	2.9(1.47,5.82)	8(2.53,25.20) **	
Primary school education	13	139	2.1(1.02, 4.19)	4.9(1.65,14.44) **	0.001
Secondary school education	9	110	2.1(0.60, 2.88)	1.1(0.42, 2.69)	
Higher education	23	133	1.00		
**Occupation**					
Government employee	14	131	1.00		
Nongovernment employee	6	47	1.9(0.69, 5.12)	4.3(1.42, 13.31) **	
Self employed	18	151	1.7(0.81, 3.65)	1.3(0.48, 3.52)	
Daily labourer	11	63	4(1.67, 10.23)	2(0.62, 6.72)	0.009
House wife	7	123	1(0.40, 2.70)	0.3(0.10, 1.23)	
Others	5	26	3.7(1.22, 11.16)	0.7(0.14, 3.01)	
**WHO clinical Stage**					
stage I	14	148	1.00	1.00	
stage II	14	124	2.1(0.96, 4.49)	4(1.33, 11.93) **	0.001
stage III	15	237	2.2(1.02,4.70)	5.3(2.02, 13.79) **	
stage IV	18	32	2.4(1.36,6.17)	7(2.51, 20.10) **	
**TB co-infection**					
No	54	503	1.00		
Yes	7	38	2.5(1.09, 5.51)	1.9(0.71, 4.99)	0.203
**OI prophylaxis**					
Yes	47	470	1.00	1.00	
No	14	71	1.8(0.94, 3.28)	3.2(1.47, 7.08) **	0.004
**Baseline CD4 count (cells/μL)**					
<200	42	290	1.00	1.00	
≥200	19	251	0.7(0.37, 1.19)	0.6(0.27, 1.28)	0.182

Note: ** shows association at p-value of 0.05 with the outcome variable.

CHR = crude hazard ratio, AHR = adjusted hazard ratio.

The raw data attached (S1 Data).

## Discussion

The purpose of this study was to determine the incidence rate and time to the development of ADRs of HAART regimen and its predictors among adult HIV patients. Accordingly, 61(10%) of the total study subjects developed ADRs. Of these events 37(60.6%) occurred in the first six months, 49(80.3%) events happened in one year period, and 12(19.65%) cases occurred in the remaining four years. This finding is supported by study done in Nigeria and Southern India, where most of ADRs occurred in the first half of the follow up periods [14, 15].

In our study the cumulative probability of surviving without developing adverse drug reaction at the end of half, one, two and end of follow up years was 0.94, 0.91, 0.89 and 0.88 respectively. This finding was higher than a study conducted in South Africa [16]. The difference might be attributed to difference in drug regimen. The majority (71%) of study participants in the current study had been taking EFV based regimen; however, 54% of patients in South Africa had taken NVP based regimen.

In this study, the rate of occurrence of adverse drug reaction was 4.3/100PY. This finding is similar with the study done in South Africa where the incidence of ADRs was 4.2/100PY [16]. However, our finding is lower than a study conducted in Northwest Ethiopia where the rate was 10.11/100PY [17]. The incidence of ADRs in our study is much lower than a study done in Southern India where the rate was 15/100PY [18]. This difference might be due to the difference in follow up time, in our case the follow up time was five years and the ADRs occurred in the first two years of the follow up period, however, the study in India was a two years follow up, which is less than half our study period, this might contributed to large rates of ADRs as most of ADRs likely to occur in the first one to three years.

In this study, HIV positive patients with no formal education and completed only primary education were about eight and four times at risk of ADRs as compared to patients attended higher level of education respectively. This finding is in line with a study done in Northern Nigeria, and Jodhpur-India where educational level of the patients was significantly associated with the time to develop ADRs [19, 20]. It might be inferred that higher educational attainment offered some protection of ADRs due to proper understanding of ARV drug adherence, good nutritional status and gave care for them.

The occupation of the patients showed association with ADRs, where nongovernmental workers were more than four times at higher risk of developing ADRs at any given time as compared to patients in governmental organizations. This finding is similar to a study done in Northern Nigeria where occupational status of the study patients significantly associated with the time to develop ADRs in HIV positive patients [19]. This might be due to poor drug adherence, as nongovernmental employees mostly pass their time in field work, they could forget to take medications, drug could stock out and they may lose / run out of pills.

The WHO clinical stage of the patients was the other important predictors of ADRs, where patients in the advanced clinical stage at the initiation of HAART were at risk of developing ADRs at any time compared to patients in clinical stage I. The find is in line with similar other studies where clients in the advanced clinical stage of the disease were more likely to get ADRs [18, 21, 22]. This might be due to the fact that those patients in the advanced stages of the disease could be unable to resist drug side effects and resulted in drug changes or hospital admissions [23]. In addition, patients in the advanced disease stage are likely to be on other medications which might result in drug interactions and overlapping toxicity between ART drugs and other medications that could result in drug changes/ hospital admissions. The other possible explanation might be poor adherence due to pill burden which resulted in poor efficacy of treatment result in drug change/ hospital admission.

Opportunistic infection (OI) prophylaxis was found to be associated with ADRs; where patients didn’t receive the prophylaxis were more than three times at risk of adverse drug reaction as compared to those clients received OI prophylaxis. This is supported by the Center for Disease Control and Prevention (CDC) report which revealed OI prophylaxis prevents adverse drug reactions [24]. The possible reason might be due to the prophylactic therapy successfully extends the occurrence of ADRs and improves the quality of life for people living with HIV.

In this study Sex, age, marital status, CD4 count and regimen type were not statistically significant predictors of development of ADRs. This finding contradicts the findings of similar studies conducted in South Africa, Northern Nigeria and Zimbabwe [16, 19, 25]. This discrepancy might be explained for possible variations of the study settings, regimen type and time gap in which the studies were conducted. The sample size of our study was relatively small, this means chance could have an effect for these factors failed to show significant association with the outcome variable.

This study has some limitations. First, because of the retrospective nature of the study some important predictors that might have association with initial regimen change in other studies, like BMI. Secondly, there might be misclassification of ADRs, poor recording and reporting of adverse drug reactions.

## Conclusion

According to this study, out of 100 HIV positive patients on HAART, five of them developed ADRs per one year of follow up, which is higher than most similar other studies, and most of the adverse drug reactions occurred within a year after initiation of HAART; three fifth (60.6%) of them were occurred in the first six months of the commencement of the treatment.

The educational status, occupation, clinical stages and OI prophylaxis therapy were important explanatory variables for the ADRs. Therefore, Federal ministry of health should promote pre education program for HIV positive patients on ART in order to minimize problems associated with health risks including ADRs. The health workers need to give attention to the educational level and provide counseling for their patients to prevent the occurrence of ADRs. Health workers should counsel about OI prophylaxis intake and strictly follow their clients whether the patient take the drug or not in order to minimize the associated ADRs in patients on HAART.

Health workers in ART clinics and hospitals in general need to give special care and attention to HIV positive patients in advanced WHO clinical stage (III and IV) to minimize the associated ADRs.

## Supporting information

S1 Data (RAR)
