# Correction: Time to development of adverse drug reactions and associated factors among adult HIV positive patients on antiretroviral treatment in Bahir Dar City, Northwest Ethiopia

**DOI:** 10.1371/journal.pone.0221608

**Published:** 2019-08-20

**Authors:** The PLOS ONE Staff

## Notice of republication

This article was republished on August 12, 2019 to correct an error in the References. The publisher apologizes for this error. Please download this article again to view the correct version.
